# Relationship between social support and fear of cancer recurrence among Chinese cancer patients: A systematic review and meta-analysis

**DOI:** 10.3389/fpsyt.2023.1136013

**Published:** 2023-03-08

**Authors:** Xianying Lu, Chenxi Wu, Dingxi Bai, Qian You, Mingjin Cai, Wei Wang, Chaoming Hou, Jing Gao

**Affiliations:** School of Nursing, Chengdu University of Traditional Chinese Medicine, Chengdu, Sichuan, China

**Keywords:** fear of cancer recurrence (FCR), social support, cancer, meta-analysis, systematic review

## Abstract

**Background:**

To quantitatively analyze the association between social support (SS) and fear of cancer recurrence (FCR) by reviewing current evidence from observational studies.

**Methods:**

A comprehensive literature search was performed in nine databases from inception to May 2022. Observational studies that used both SS and FCR as study variables were included. Regression coefficient (β’) and correlation coefficient (*r*) were calculated with R software. Subgroup analysis was utilized to investigate the degree of the relationship between SS and FCR as well as the impact of various forms of SS on FCR in cancer patients.

**Results:**

Thirty-seven studies involving 8,190 participants were identified. SS significantly reduced FCR risk [pooled β’ = –0.27, 95% confidence interval (CI) = –0.364 to –0.172], with moderate negative correlations (summary *r* = –0.52, 95% CI = –0.592 to –0.438). Meta-regression and subgroup analysis showed that types of cancer and study type were the source of heterogeneity. However, types of SS [actual SS, perceived social support (PSS), and others], source of actual SS, and source of PSS were not significant moderators.

**Conclusion:**

To the best of our knowledge, this is the first systematic review and meta-analysis to quantitatively investigate the association between SS and FCR in Chinese cancer patients using β’ and *r* coefficients. The results re-emphasized that social workers should enhance the use of SS by cancer patients and establish a sound SS system by either implementing more relevant research or developing targeted policies. Based on meta-regression and subgroup analyses, moderators of the association between SS and FCR should also be studied closely as they may help identify patients in need. In addition, longitudinal research, as well as mixed research, should be conducted to more comprehensively explore the relationship between SS and FCR.

**Systematic review registration:**

https://www.crd.york.ac.uk/prospero, identifier CRD42022332718.

## Introduction

Cancer is a major public health problem worldwide and the second-leading cause of death after cardiovascular disease, and it is likely to become the leading cause of death by 2060 (1). As one of the most common chronic diseases, cancer has the characteristics of high morbidity, high mortality, and high recurrence. Fortunately, the survival rate and survival time of cancer patients have been improved significantly owing to increased public health awareness, early diagnosis, and advancement in medical technology (2). The 5 years survival rate for breast cancer is 68.1–93.2% in the United States (3), while the 5 years survival rates for breast cancer, cervical cancer, and thyroid cancer in China are 82.0, 59.8, and 84.3%, respectively (4–6). Cancer recurrence and metastasis have remained a challenge for modern medical science and a source of worry and threat for cancer patients.

Fear of cancer recurrence (FCR), a negative psychological experience that persists in cancer patients during and after the treatment of the disease, is defined as the “fear, worry, or concern related to the possibility of cancer recurrence or progression in the same organ or other parts of the body” (7). The incidence of FCR ranges from 33 to 96%, which may appear immediately after diagnosis and persist for many years (8). Schapira et al. (9) studied FCR trajectories in breast cancer survivors and confirmed that although FCR improved over time in some breast cancer survivors, it remained severe in approximately one-third of breast cancer survivors for up to 5 years after diagnosis. Simard et al. (10) reported that 39–97% of patients had a lower level, 22–87% had an intermediate level, and ≤15% had a higher level of FCR. FCR is a normal psychological response to stress in patients. When negative stimulation or stress is moderate, the body can make necessary adjustments for malignancies and promote healthy behaviors (11). However, if the stress exceeds the normal range, it may lead to sleep disorders (12) and dysfunction (13). In addition, FCR can aggravate patients’ anxiety and depression, seriously affecting their wellbeing and quality of life, which in turn affects the efficacy of chemotherapy drugs and increases the incidence of adverse drug reactions (14, 15). Patients with high levels of FCR may undergo an excessive physical examination and hypervigilance, taking any symptoms such as pain and chest tightness as signs of disease aggravation (16), and in extreme cases may result in serious psychiatric disorders such as somatic symptom disorder or post-traumatic stress disorder (PTSD) (17–19). Several studies have also confirmed that patients with high levels of FCR are more likely to overuse healthcare resources (e.g., increased frequency of unplanned visits, overdoses, requests for tests beyond clinical indications, or hospital refusals), thereby increasing the cost of national health care and family financial burdens (20, 21). A recent meta-analysis by Williams et al. (22) confirmed that rational control of excessive medical behavior related to FCR may bring significant financial benefits. In conclusion, the prevention and control of FCR not only improves physical and mental health, and thus reduces financial stress for the patients, but also helps to ease the pressure on the national health care system.

FCR can be affected by multiple factors, including social support (SS). Cancer is regarded as a traumatic and stressful event, and cancer patients often undergo a series of physical, emotional, and social changes following diagnosis, treatment options, and side effects of treatment, which may lead to feelings of inadequacy. Therefore, having a strong SS is critical to a successful post-cancer psychological adjustment. SS is a general term that refers to various services provided by social networks to individuals that can improve mental health or lessen psychological problems, which include sub-structures such as actual SS and perceived social support (PSS) (23). Since these two kinds of SS were the most common measurement forms in China, in our study, we would apply this concept of social support. Actual SS in cancer patients is measured using a Social Support Rating Scale (SSRS) and encompasses subjective support, objective support, and support availability (24). Subjective support, also known as the subjective experience or emotional support of an individual, is spiritual support such as respect, understanding, and encouragement from others in the social network to which the individual has access. Objective support is visual assistance and support, including material assistance, presence, and engagement in group connections. Support availability refers to the extent to which the individual seeks help from others or society, including facets such as how the patient requests assistance, how he or she confides in others when in need, and whether or not the patient engages in group activities (24). PSS in cancer patients, measured by the Perceived Social Support Scale (PSSS), refers to the extent to which individuals can subjectively feel, understand, and comprehend various types of SS from family, friends, or others (25). Cheng (26) suggested that assessing different aspects of SS may help to explore the impact of different types of SS on FCR. Meanwhile, Thompson et al. (27) showed that psychological problems in cancer survivors are closely related to social factors, and SS plays an important role in the quality of life and health outcomes after breast cancer diagnosis and treatment. Chen and Geng (28) highlighted that effective SS enhances cancer patients’ psychological resilience and hope. The theory of SS and stress-buffering holds that more SS can protect individuals under stress and improve their ability to deal with stressful events (29). In addition, Niu et al. (30) investigated 342 breast cancer patients and found that SS was an independent predictor of FCR.

It has been proven that social factors contribute more to changes in FCR than demographic and disease-related factors (31); thus, it is vital to comprehend FCR among cancer patients from an SS perspective to develop preventive measures and lessen the detrimental consequences of FCR in cancer patients. Chinese cancer patients receive emotional or economic support from family, friends and other aspects, and FCR decreases with an increase in SS among Chinese cancer patients (32–34), this is consistent with the findings of quantitative studies of SS and FCR for Indonesian gynecological cancer (35) and Asian-American breast cancer (36) cancer patients. However, a positive correlation between SS and FCR has been reported among South Asian breast cancer survivors in a qualitative research (37) and Iranian cancer patients in a quantitative study (38). In a quantitative study, Thewes et al. (39) found no correlation between SS and FCR among Australian breast cancer patients. These studies indicated that FCR might be related to SS systems from different countries. The finding of one country cannot be generalized to other countries or regions, and it is unclear whether existing plans and interventions aimed at reducing FCR are appropriate or effective for cancer survivors in China. Hence, a comprehensive analysis of the correlation between SS and FCR in China is of great significance. China has a high incidence of cancer, accounting for about 50% of all cases in Asia (40). This is surprising given that the number of cancer survivors is continuously increasing due to the large population and high incidence as well as the high FCR detection rate in cancer patients (30, 41, 42). Although FCR among cancer patients has gradually gained increasing attention in China owing to the increased detection rate of FCR in cancer patients, FCR is still not fully understood by Chinese academics and the medical community because research on FCR among cancer survivors started quite later in China compared with Western nations (such as the United States, Netherlands, and Canada) (43). At present, several studies have investigated the level of FCR from an SS perspective among Chinese cancer patients, yielding mixed results with no consensus on the extent. Specifically, some studies have reported a relatively large negative correlation (32–34), while others have found a small negative correlation (41, 44), the degree of correlation varied widely (r ranged from –0.144 to –0.804). Because all participants were Chinese and the cultural differences caused by homogeneous ethnic backgrounds were small, the heterogeneity due to ethnic differences can be ignored when performing meta-analyses. Therefore, given the mixed results in the Chinese population and the lack of a systematic review evaluating the relationship between SS and FCR among Chinese cancer patients, it is reasonable to conduct a meta-analysis to determine the degree of connection between SS and FCR among Chinese cancer patients. This review aimed to bridge this knowledge gap and offer a Chinese perspective for the management of social workers and medical staff around the world, thus giving a scientific basis for more targeted interventions to mitigate, prevent, or control FCR among cancer patients, which might be of great benefit to individuals, families, society, and the nation.

Research on the same topic producing quite different and sometimes contradictory conclusions affects the reliability of related studies, is unlikely to give a clear future research direction, and may even cause study selection bias in systematic reviews and meta-analyses, thus making it difficult for social workers and medical staff to value their policy implications and suggestions, which may harm the theoretical and practical development of the health service security system for cancer patients. Using meta-analysis, the outcomes of studies can be statistically combined to obtain the overall effect quantity. However, no meta-analysis has been conducted on the association between SS and FCR in China at present. Herein, we conducted a meta-analysis, with regression coefficient (β’) and correlation coefficient (*r*) as the evaluation basis, to deeply explore the correlation between SS and FCR among Chinese cancer patients. Furthermore, subgroup analysis was used to analyze the moderating effects of the sample size, region, follow-up, types of cancer, publication year, and the association of FCR and types of SS (actual SS, PSS, and each dimension of SS), to provide a foundation for future research on the proper use of an SS system to improve FCR in cancer patients.

## Methods

This systematic review was conducted following the Preferred Reporting Items for Systematic Reviews and Meta-Analyses (PRISMA) statement guidelines and was registered on the International Prospective Register of Systematic Reviews (PROSPERO) (registration number: CRD42022332718). Because this was a review with meta-analysis, ethical approval was not required.

### Search strategy

A comprehensive literature search was conducted in nine databases, including PubMed, Embase, Web of Science, Cochrane, China National Knowledge Infrastructure (CNKI), VIP Database for Chinese Technical Periodicals (VIP), Wanfang Data, Chinese Biomedical (CBM), and Cumulative Index to Nursing and Allied Health Literature (CINAHL), from the inception to May 2022. Key terms, including “neoplasm/tumor*/cancer/malignancy/carcinoma” AND “fear/worry/concern/uncertainty/fear of cancer recurrence” AND “recurrence/relapse/progress*/exacerbation/return” AND “social support/perceived social support,” were used without date restrictions. In addition, reference lists of the retrieved articles were manually checked to identify additional relevant studies. The specific search strategy is shown in Supplementary Appendix A.

### Inclusion and exclusion criteria

The inclusion criteria were as follows: (a) Chinese cancer patients aged ≥18 years, having received treatments such as surgery, chemotherapy, or radiotherapy; (b) observational study; (c) reported the relationship between FCR and SS, such as a correlation coefficient (*r*), or regression coefficient (β’); (d) used a validated scale to assess FCR, including Fear of Progression Questionnaire-Short Form (FoP-Q-SF), Concerns About Recurrence Scale (CARS), Fear of Cancer Recurrence Inventory (FCRI) and Fear of Progression Questionnaire (FoP-Q), have been proved to be valid through reliability and validity tests. The exclusion criteria were as follows: (a) studies that were not in English or Chinese language; (b) studies with incomplete data or data that could not be analyzed.

### Date extraction

Two reviewers (LXY and WCX) independently screened the literature and extracted data after documents being imported into Endnote X9. The process of literature screening was as follows: exclude the duplicate studies; read the titles and abstracts to exclude clearly irrelevant articles (unrelated to our outcome of interest) based on the inclusion criteria; and read the full text to further determine their suitability. The following data were extracted with a predefined data extraction form (Table 1) to ensure the accuracy of the collected data by stringently following the inclusion and exclusion criteria as mentioned above, and sequential exclusion of the unsuitable studies, which include the following information: study characteristics (first author, publication year, region, study design, and duration of follow-up), characteristics of the participants (age, sex, sample size, types of cancer, and instruments used to measure FCR and SS), and effective outcome data [Pearson or Spearman correlation coefficient (*r*) and regression coefficient (*ß’)* between SS and FCR]. Any disagreements in data were resolved by a third party (BDX).

**TABLE 1:** Data-collection form.

First author	Publication year	Age	Study design 1. Sample size (male/female) 2. Random sampling 3. Region of China 4. Types of cancer 5. Follow-up 6. Measuring tool 6.1 FCR measure 6.2 SS measure	Risk of bias assessment	Main findings (*r*, or β ’) 1. Overall SS and FCR 2. Types of SS and FCR 3. Source of SS and FCR 4. SS and FCR dimension

Pearson correlation coefficient (*r*_*p*_) was used for the meta-analyses. Original studies were transformed before analysis and the published Spearman correlation coefficient (*r*_*s*_) was converted into Pearson correlation coefficient (*r*_*p*_), The conversion formula is as follows. Since the standard error (SE) depends on the value of the correlation coefficient, a Fisher transformation was used to convert each correlation coefficient.


(1) $$r_{p}=2\,\text{sin}\,\left(r_{s},\frac{\pi }{6}\right)$$

### Risk of bias assessment

Two reviewers (LXY and WCX) independently evaluated the quality of included observational studies using Joanna Briggs Institute (JBI) critical appraisal tools (Supplementary Appendix B). Each study was assessed according to nine items with a full score of nine scores and each item with four answers, respectively “Yes,” “No,” “Unclear,” and “Not applicable.” The item would be scored one if the answer was “Yes.” Otherwise, it would be scored zero. Those with scores of ≥6 were identified as high quality. Any disagreements in data were resolved by a third party (BDX).

### Statistical analysis

The meta-analysis was performed using the “meta” package in R version 4.1.3. Heterogeneity in systematic reviews was generally described as clinical, methodological, and statistical heterogeneity (the result of clinical and/or methodological diversity among individual studies) (46), and was assessed by the *I*^2^ statistic and *Q*-test (*P*-value); if *P* > 0.10 and *I*^2^ < 50%, a fixed effects model was chosen; otherwise, a random effects model was adopted. When heterogeneity occurred, meta regression, subgroup analysis, and sensitivity analysis were performed to assess the source of heterogeneity. Among them, subgroup analysis was used to determine significant clinical heterogeneity and methodological heterogeneity, and could only be performed for only one covariate that was a categorical variate at a time. In contrast, meta-regression, which could reflect the relationship between one or more covariates (could be categorical or continuous variables) and outcome variables by establishing a regression equation, was performed to investigate the sources and size of heterogeneity among individual studies. The selected covariates could be some characteristics among the study or trial level, such as study design, intervention dose, administration route, treatment duration, gender, age, ethnicity of the patient, and research sample size; or they can be the combined characteristics of cases included within a single study, such as the average age and average height of patients (46, 47). The meta-regression criteria were (1) *P* ≤ 0.10 for the *Q* test or *I*^2^ greater than 50%; (2) *P* ≥ 0.05 for Egger’s test; (3) and response variables reported in at least 10 studies. To ensure each covariate was scientifically sound, the covariates should be identified based on clinical assumptions and biology (46). Therefore, based on the above literature (46, 47) and previous related studies (48–78), we assumed that the heterogeneity might arise from the age, gender (male and female), sample size (<200 and ≥200), types of cancer (breast cancer group, mixed-type group, and other-type group), publication year, region (north and south), follow-up, random sampling, instruments used to measure FCR and SS, and source of SS. In this study, meta-regression was performed to explore whether the differences in categorical covariates such as SS measurement, FCR measurement, types of cancer, follow-up, and region, whereas sample size and publication year were used as continuous covariates, when covariates were statistically significant, with *P* ≤ 0.05. Furthermore, we performed a subgroup analysis of those variables and a leave-one-out method by iteratively removing the included study of sensitivity analysis. Then, sensitivity analysis was also used to detect the stability of the results. Meanwhile, funnel plots, Begg’s test, and Egger’s test were used to detect publication bias. A correlation coefficient (*r*) varies between –1 and 1, | *r*| < 0.2 implies no correlation; 0.2 < | *r*| < 0.4 suggests a weak correlation; 0.4 < | *r*| < 0.6 indicates a moderate correlation; 0.6 < | *r*| < 0.8 signifies a strong correlation; 0.8 < | *r*| denotes excellent correlation.

## Results

### Study selection

**FIGURE 1:**
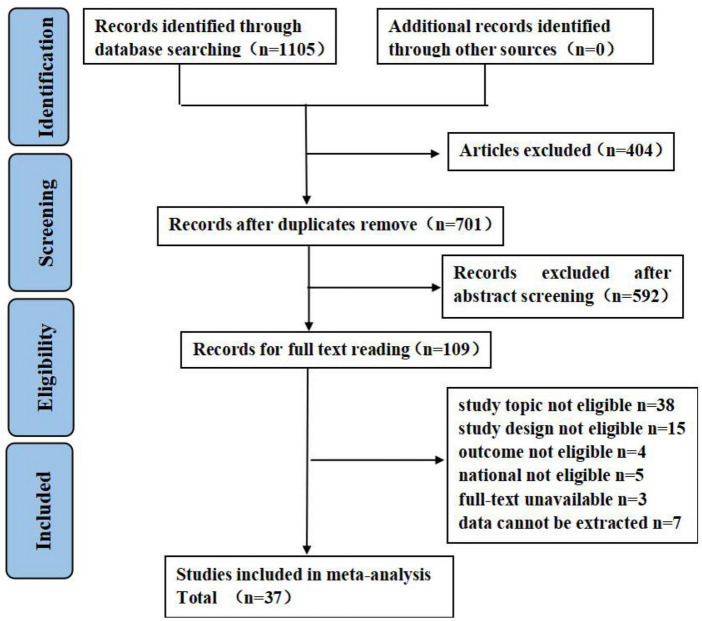
Flow diagram of literature search. Adapted from Page et al. (45) licensed under CC-BY 4.0.

A total of 1,105 studies were retrieved, of which 404 studies were excluded due to duplication and 592 studies were excluded after an initial screening based on titles and abstracts. A total of 109 studies were selected for full-text screening, of which 37 articles met the eligibility criteria. Details on study selection the process are shown in Figure 1. Results of the quality assessment are shown in Supplementary Appendix C.

### Study characteristics

A total of 37 studies were conducted across 12 different provinces in China, involving 8,190 individuals (ranging from 77 to 857), of which 5,892 were females and 2,298 were males. Patients were aged between 18 and 83 years; however, two articles did not report the median or mean age. The publication date of articles ranged from 2018 to 2022, where four articles were published in 2018, 10 in 2019, 11 in 2020, 9 in 2021, and 3 in 2022. All publications were cross-sectional studies except a study by Li et al. (32). The Fear of Progression Questionnaire-Short Form (FoP-Q-SF) was the most frequently used to measure FCR status (*n* = 27). Social Support Rating Scale (SSRS, *n* = 20) and Perceived Social Support Scale (PSSS, *n* = 14) were the most frequently used to measure SS, which indicated that over half (20/37, 54.05%) of studies examined specific SS from subjective support, objective support, and support availability and 14 studies examined perceived SS in cancers patients from multiple sources (e.g., family, friend, and other). Two studies reported the *ß’* coefficient, 17 studies reported the *r* coefficient, and 18 studies reported both. Detailed characteristics of included studies are shown in Table 2.

**TABLE 2:** The characteristics of 37 studies included in this meta-analysis.

References	Publication year	Types of cancer	Provinces of China	Sample (male/female)	Age	FCR measure	SS measure	Main finding (*r*, or β’)
Zhong et al. (48)	2020	Glioma	Jiangsu	128 (76/52)	27∼69 (42.54 ± 5.54)	FoP-Q-SF	PSSS	Correlation: PSSS total, family support, friend support, other support (*r* = –0.505, –0.428, –0.599, –0.485)
Li et al. (49)	2019	Nasopharyngeal	Guangdong	210 (153/57)	37.14 ± 9.28	FoP-Q-SF	PSSS	*Correlation: PSSS total, family support, friend support (*r* = –0.397, –0.411, –0.356)
								*Prediction: PSSS total (β’ = –0.234)
Guan et al. (50)	2020	Nasopharyngeal	Guangdong	180 (85/95)	35∼75	FoP-Q-SF	PSSS	*Prediction: PSSS total (β’ = –0.168)
Zeng et al. (51)	2021	Nasopharyngeal	Guangdong	140 (82/58)	—	FCRI	SSRS	Correlation: SSRS total, subjective support, objective support, support availability (*r* = –0.532, –0.254, –0.263, –0.36)
Yuan et al. (33)	2021	Laryngeal	Anhui	77 (44/33)	35∼62 (46.35 ± 5.12)	FoP-Q-SF	SSRS	*Correlation: SSRS total, subjective support, objective support, support availability (*r* = –0.724, –0.632, 0.732, –0.812)
Ye et al. (52)	2022	Laryngeal	Anhui	146 (42/104)	≥18	FoP-Q-SF	SSRS	*Correlation: SSRS total, subjective support, objective support, support availability (*r* = –0.334, 0.271, –0.328, –0.298)
								Prediction: SSRS total (β’ = –0.13)
Lin (53)	2021	Lung	Shandong	260 (184/76)	≥60	FoP-Q	PSSS	Correlation: PSSS total, family support, friend support, other support (*r* = –0.682, –0.48, –0.623, –0.715)
								Prediction: PSSS total (β’ = –0.799)
Li et al. (32)	2021	Lung	Shandong	81 (50/31)	52∼71 (60.53 ± 2.36)	FCRI	SSRS	*Correlation: SSRS total (*r* = –0.804)
Cui and Lin (54)	2021	Liver	Fuzhou	308 (218/90)	26∼72 (56.22 ± 9.22)	FoP-Q-SF	SSRS	*Correlation: SSRS total, subjective support, objective support, Support availability (*r* = –0.436, –0.33, –0.452, –0.409)
								*Prediction: SSRS total (β’ = –0.268)
Deng et al. (55)	2019	Liver	Guangdong	154 (109/45)	26∼73 (56.22 ± 9.23)	FoP-Q-SF	SSRS	*Correlation: SSRS total, subjective support, objective support, support availability (*r* = –0.435, –0.329, –0.451, –0.408)
								Prediction: SSRS total (β’ = –0.267)
Cheng (56)	2020	Liver	Liaoning	220 (167/53)	58.15 ± 9.89	FoP-Q-SF	SSRS	Correlation: SSRS total, subjective support, objective support, support availability (*r* = –0.586, –0.460, –0.504, –0.578)
								Prediction: SSRS total (β’ = –0.164)
Chen et al. (57)	2019	Lymphoma	Guangdong	142 (84/58)	≥18	FoP-Q-SF	SSRS	*Correlation: SSRS total, subjective support, objective support, Support availability (*r* = –0.362, –0.385, –0.396, –0.313)
								Prediction: SSRS total (β’ = –0.184)
Zhang (58)	2018	Lymphoma	Henan	279 (181/98)	18∼83 (48.05 ± 17.03)	FoP-Q-SF	PSSS	Correlation: family support, friend support (*r* = –0.334, –0.345)
								*Correlation: family support, friend support (*r* = –0.359, –0.369)
Hu et al. (59)	2022	Myeloma	Guangdong	127 (0/127)	28∼80 (58.09 ± 9.52)	FoP-Q-SF	SSRS	Correlation: SSRS total, subjective support, objective support, support availability (*r* = –0.287, –0.342, –0.137, –0.061)
								Prediction: SSRS total (β’ = –0.105)
Luo et al. (60)	2021	Colorectal	Guangdong	146 (97/49)	≥18	FoP-Q-SF	PSSS	Correlation: PSSS total, family support, friend support (*r* = –0.34, –0.3, –0.28)
								Prediction: PSSS total (β’ = –0.34)
Zhao (61)	2020	Colorectal	Henan	314 (195/119)	59.31 ± 10.27	FCRI	MSPSS	Correlation: MSPSS total (*r* = –0.597)
Gao (62)	2021	Colorectal	Zhejiang	203 (115/88)	67.54 ± 8.45	FoP-Q-SF	PSSS	Correlation: PSSS total, family support, friend support (*r* = –0.745, –0.452, –0.672)
Xu et al. (63)	2020	The upper gastrointestinal tract	Jiangsu	348 (248/100)	52.50 ± 7.90	FoP-Q-SF	SSRS	Correlation: SSRS total, subjective support, objective support, support availability (*r* = –0.372, –0.357, –0.228, –0.288)
He et al. (44)	2020	Stomach	Zhejiang	120 (68/52)	56∼75 (65.7 ± 9.2)	FoP-Q-SF	PSSS	Correlation: PSSS total, family support, friend support, other support (*r* = –0.144, –0.068, –0.184, –0.125)
Liao et al. (64)	2018	Malignancies	Sichuan	126 (0/126)	≥18	FoP-Q-SF	PSSS	Correlation: PSSS total (*r* = –0.721)
								Prediction: PSSS total (β’ = –0.158)
Zhang et al. (65)	2019	Gynecological malignancies	Jiangsu	187 (0/187)	20∼73 (53.3 ± 12)	FoP-Q-SF	CSS	Correlation: SSRS total, subjective support, objective support (*r* = –0.314, –0.372, –0.221)
								Prediction: SSRS total (β’ = –0.314)
Zhai et al. (66)	2020	Cervical	Henan	100 (0/100)	31∼69 (51.82 ± 9.78)	FoP-Q-SF	SSRS	Prediction: SSRS total (β’ = –0.356)
Lai and Li (67)	2019	Cervical	Guangdong	140 (0/140)	26∼67	FoP-Q-SF	SSRS	Correlation: SSRS total (*r* = –0.44)
								Prediction: SSRS total (β’ = –0.365)
Ma and Li (68)	2018	Ovarian	Tianjin	157 (0/157)	≥18	FCRI	SSRS	Correlation: SSRS total (*r* = –0.44)
								Prediction: SSRS total (β’ = –0.226)
Zhong (69)	2020	Cervical	Guangdong	137 (0/137)	20∼49 (42.47 ± 1.76)	FoP-W-SF	SSRS	Correlation: subjective support, objective support, support availability (*r* = –0.642, –0.306, –0.104)
Li et al. (41)	2019	Breast	Shandong	364 (0/364)	NA	FoP-Q-SF	PSSS	Correlation: PSSS total, family support, friend support, other support (*r* = –0.145, –0.075, –0.171, –0.121)
Ye et al. (70)	2019	Breast	Guangdong	180 (0/180)	28∼69	FoP-Q-SF	SSRS	Correlation: SSRS total, subjective support, objective support, support availability (*r* = –0.429, –0.509, –0.291, –0.464)
Ren (43)	2021	Breast	Unclear	857 (0/857)	20∼79 (47.18 ± 9.86)	FCRI-SF	SSRS	Correlation: SSRS total (*r* = –0.511)
								Prediction: SSRS total (β’ = –0.118)
Zhang et al. (71)	2018	Breast	Henan	270 (0/270)	≥18	FoP-Q-SF	PSSS	Correlation: PSSS total (*r* = –0.464)
								Prediction: PSSS total (β’ = –0.227)
Guo (72)	2020	Breast	Hebei	325 (0/325)	≥18	FoP-Q-SF	PSSS	Correlation: PSSS total, family support, friend support (*r* = –0.29, –0.158, –0.337)
Zhou (73)	2020	Breast	Jiangsu	186 (0/186)	30∼73 (49.34 ± 8.4)	FoP-Q-SF	PSSS	Correlation: PSSS total, family support, friend support, other support (*r* = –0.317, –0.329, –0.118, –0.106)
								Prediction: PSSS total (β’ = –0.197)
Niu (34)	2018	Breast	Jiangsu	342 (0/342)	30∼81 (51.46 ± 10.5)	FoP-Q-SF	SSRS	Correlation: SSRS total, subjective support, objective support, Support availability (*r* = –0.63, –0.549, –0.448, –0.521)
Ban et al. (74)	2021	Breast	Liaoning	244 (0/244)	54.3 ± 10.5	FoP-Q-SF	MSPSS	Correlation: MSPSS total (*r* = –0.239)
Yu et al. (75)	2022	Breast	Liaoning	231 (0/231)	31∼82 (67.54 ± 8.45)	FCRI-SF	PSSS	Correlation: PSSS total, family support, friend support, other support (*r* = –0.38, –0.36, –0.31, –0.34)
Ren (76)	2020	Breast	Tianjin	257 (0/257)	≥18	CARS	SSRS	Correlation: SSRS total, subjective support, objective support, support availability (*r* = –0.472, –0.38, –0.267, –0.447)
								Prediction: SSRS total (β’ = –0.232)
Zhang and Zhang (77)	2019	Breast	Hunan	312 (0/312)	19∼76	FoP-Q-SF	SSRS	Correlation: SSRS total (*r* = –0.472)
								Prediction: SSRS total (β’ = –0.11)
Xing et al. (78)	2019	Mixed	Shandong	192 (100/92)	27∼81 (40.21 ± 5.8)	FCRI	SSRS	Correlation: SSRS total, subjective support, objective support, support availability (*r* = –0.562, –0.08, 0.335, –0.174)

FoP-Q-SF, Fear of Progression Questionnaire-Short Form; FoP-W-SF, Fear of Progression Worry-Short Form; FoP-Q, Fear of Progression Questionnaire; CARS, Concern About Recurrence Scale; FCRI, Fear of Cancer Recurrence Inventory; FCRI-SF, Fear of Cancer Recurrence Inventory-Short Form; PSSS, Perceived Social Support Scale; SSRS, Social Support Rating Scale; MSPSS, Multi-Dimensional Scale of Perceived Social Support; CSS, Couple Support Scale. *The correlation coefficient r or the standard regression coefficient β’ was extracted at the follow-up of cancer patients.

### Meta-analysis

**FIGURE 2:**
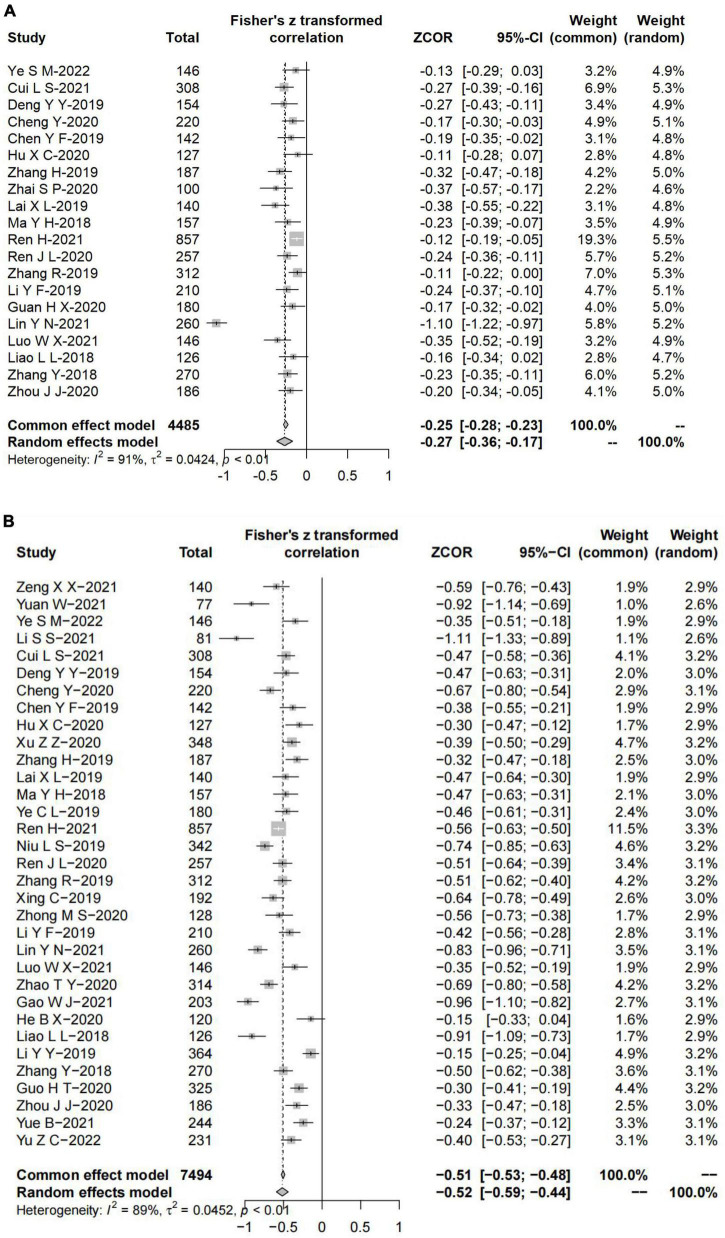
Forest plots of the pooled β’ **(A)** and summary *r* coefficients **(B)**, respectively.

Twenty studies reported the *ß’* coefficient, pooled *ß’* was –0.27 (95% confidence interval (CI) = –0.36 to –0.17), with substantial heterogeneity (*I*^2^ = 91%, *P* < 0.01) (Figure 2A). Thirty-three studies reported the *r* coefficient between FCR and SS. The result demonstrated that FCR was negatively correlated with SS (summary *r* = –0.52, 95% CI = –0.59 to –0.44), with substantial heterogeneity (*I*^2^ = 89%, *P* < 0.01) (Figure 2B).

### Meta-regression analysis

The SS measurement, FCR measurement, types of cancer, follow-up, sample size, publication year, and region were chosen as covariates. However, the above covariates showed that there was no significant effect on the relationship between SS and FCR based on the result of pooled *ß’* and *r* coefficients (*P* > 0.05) (Table 3).

**TABLE 3:** Meta-regression analysis of the summary *r* coefficient between social support (SS) and fear of cancer recurrence (FCR).

Variable	β ’			*r*		
	***P***	***I*^2^ (%)**	**tau^2^**	***P***	***I*^2^ (%)**	**tau^2^**
SS measurement	0.207	89.66	0.041	0.279	90.81	0.045
FCR measurement	0.062	88.18	0.037	0.122	90.19	0.043
Types of cancer	0.269	89.55	0.042	0.123	90.40	0.043
Follow-up	0.650	90.45	0.044	0.272	90.96	0.045
Sample size	0.679	89.38	0.045	0.657	90.61	0.047
Publication year	0.366	89.94	0.043	0.628	91.08	0.047
Regions of China	0.112	88.04	0.040	0.684	90.76	0.049

### Subgroup analysis of the association between SS and FCR

As shown in Table 4, thirty-three studies examined the association between overall SS and FCR, of which 18 studies measured by SSRS examined the specific relationship between SS from subjective support (*n* = 15), objective support (*n* = 15), and support availability (*n* = 14) and FCR, 12 studies measured by PSSS examined the specific association between perceived SS from family (*n* = 12), friend (*n* = 12), and other (*n* = 6) and FCR. Because SSRS and PSSS were the most frequently used to measure SS, subgroup analyses were undertaken to explore the effect size between types of SS (different measurement tools) and source of SS and FCR. FoP-Q-SF (*n* = 24) and CARS (*n* = 5) were the most common tools to measure FCR. However, most literature only reported the relationship between SS and total FCR score, and rarely reported the relationship between SS and FCR dimension, In addition, FoP-Q-SF includes 12-item in two dimensions (physical health dimension and social-family dimension) with the total score of 12–60 points, and CARS included 29-item in five dimensions (overall fear level, femininity concerns, health concerns, death concerns, role concerns) with a total of 4–124 points. Due to the different dimensions of the two scales and significant differences between the content of the dimensions. So subgroup analysis of the relationship between SS and FCR could not be performed according to the dimension of FCR.

**TABLE 4:** Subgroup analysis of the summary *r* coefficient between social support (SS) and fear of cancer recurrence (FCR).

Moderators	*N*	Sample size	Summary *r* (95%CI)	*P* ^a^	Heterogeneity		
					***I*^2^ (%)**	**Q**	***P* ^b^**
SS measurement	–	–	–	0.581	–	–	–
SSRS	18	4180	0.55 (–0.63, –0.46)	–	80.1	85.29	<0.01
PSSS	12	2569	–0.49 (–0.64, –0.33)	–	93.7	174.02	<0.01
Others	3	745	–0.42 (–0.69, –0.15)	–	93.5	30.82	<0.01
Source of SSRS	–	–	–	0.553	–	–	–
Subjective support	15	2957	–0.42 (–0.51, –0.33)	–	81.1	73.93	<0.01
Objective support	15	2957	–0.34 (–0.47, –0.20)	–	91.1	156.45	<0.01
Support availability	14	2770	–0.42 (–0.55, –0.28)	–	88.2	109.84	<0.01
Source of PSSS	–	–	–	0.676	–	–	–
Family support	12	2719	–0.33 (–0.42, –0.24)	–	81.8	60.6	<0.01
Friend support	12	2719	–0.40 (–0.53, –0.27)	–	90.5	115.89	<0.01
Other support	6	1289	–0.36 (–0.61, –0.10)	–	95.7	116.51	<0.01
FCR measurement	–	–	–	0.100	–	–	–
FoP-Q-SF	24	5005	–0.47 (–0.56, –0.38)	–	89.2	212.63	<0.01
FCRI	5	884	–0.69 (–0.89, –0.49)	–	82	22.19	<0.01
Others	4	1605	–0.58 (–0.75, –0.4)		88.1	25.15	<0.01
Types of cancer	–	–	–	0.047*	–	–	–
Breast	11	3568	–0.43 (–0.53, –0.33)	–	89.7	97.13	<0.01
Mixed	1	192	–0.64 (–0.78, –0.49)	–	—	0	—
Others	21	3734	–0.56 (–0.66, –0.45)	–	89.1	183.27	<0.01
Follow-up	–	–	–	0.348	–	–	–
Yes	6	972	–0.62 (–0.85, –0.38)	–	88.8	44.73	<0.01
No	27	6522	–0.50 (–0.58, –0.42)	–	89.8	255.97	<0.01
Sample size	–	–	–	0.886	–	–	–
<200	17	2429	–0.51 (–0.63, –0.39)	–	85.1	107.04	<0.01
≥200	16	5065	–0.52 (–0.63, –0.42)	–	92.9	192.88	<0.01
Random sampling	–	–	–	0.342	–	–	–
Yes	1	857	–0.56 (–0.63, –0.50)	–	—	0	<0.01
No	32	6637	–0.51 (–0.59, –0.43)	–	89.6	298.11	<0.01
Regions of China	–	–	–	0.685	–	–	–
North	12	2915	–0.54 (–0.68, –0.39)	–	92.9	154.78	<0.01
South	20	3722	–0.50 (–0.60, –0.41)		86.7	143.16	<0.01

*^a^P*-value for the between-subgroup difference. *^b^P*-value for the heterogeneity within subgroups by Q test; *P < 0.05. Bold values mean that the Pa (0.047) is < 0.05.

The summary *r* coefficient between SS and FCR showed no significant difference when stratified by SS and FCR measurement, source of SSRS and PSSS, follow-up, sample size, random sampling, and the region (all with *P*^a^** > 0.05), but all showed a statistically significant negative correlation within each group (all with *P^b^* < 0.05). Additionally, types of cancer were related to the degree of correlation (*P^a^* = 0.047). Thirty-seven studies were divided into the breast cancer group, mixed-type group (subjects containing two or more types of cancer), and other-type group (glioma, nasopharyngeal, laryngeal, lung, liver, lymphoma, myeloma, colorectal, the upper gastrointestinal tract, stomach, malignancies, gynecological malignancies, cervical, and ovarian) based on the cancer site. Results of the mixed-type group yielded a higher negative correlation than the other two groups (mixed-type group: summary *r* = –0.64, 95% CI = –0.78 to –0.49; other-type group: summary *r* = –0.56, 95% CI = –0.66 to –0.45, *P* < 0.01; breast cancer group: summary *r* = –0.43, 95% CI = –0.53 to –0.33, *P* < 0.01).

Sensitivity analysis was conducted following the statistical heterogeneity of the above subgroup analysis (all with *I^2^* > 50%). After excluding a study by Li et al. (41), Lin (53), respectively, heterogeneity reduced from 82 to 72% for family support and 96 to 83% for other support, heterogeneity may be caused by different sources of PSSS. Concerning the follow-up, it is found that heterogeneity reduced from 89 to 75% after eliminating a study by Li et al. (32), which may be because this study was a longitudinal study (Supplementary Appendix D).

### Sensitivity analysis

Sensitivity analysis was conducted by omitting each study in turn and recalculating pooled β’ and *r* coefficients to assess the robustness of our findings. The results showed no significant change, indicating that the results were stable (Supplementary Appendix E).

### Publication bias

Funnel plots of pooled *ß’* and *r* coefficients were distributed symmetrically (Supplementary Appendix F), and the Egger’s test (*P* = 0.497 and 0.670 for pooled β’ and *r* coefficients, respectively) and the Begg’s test (*P* = 0.495 and 0.642 for pooled β’ and *r* coefficients, respectively) showed no statistically significant *P*-values, suggesting that there was no significant publication bias.

## Discussion

Psycho-social elements can influence physical sickness and quality of life, leading to the development of the bio-psycho-social model. A good SS, which is a crucial external resource for cancer patients, cannot only help patients maintain a positive emotional experience but also act as a psychological stress buffer, prompting patients to face the disease more optimistically and actively cooperate with healthcare professionals during treatment and follow-up, thereby reducing and controlling FCR levels in patients (27). This study aimed to investigate the degree of the relationship between SS and FCR as well as the impact of various covariates on SS and FCR in cancer patients using meta regression, subgroup analysis, and sensitivity analysis.

To the best of our knowledge, this is the first systematic review and meta-analysis to quantitatively investigate the association between SS and FCR in Chinese cancer patients using β’ and *r* coefficients. Our results indicated that the SS among Chinese cancer patients was negatively associated with a reduced risk of FCR (pooled *ß’* = –0.27) and they had moderate negative correlations (summary *r* = –0.52), showing that FCR levels in Chinese cancer patients dropped with increasing SS levels, and SS had a moderately predictive influence on FCR. The Joanna Briggs Institute score ranged from 6 to 7 points, indicating that the quality of the included studies was high. The sensitivity analysis results were robust, suggesting that the pooled analysis of correlation coefficients was reliable and convincing. Egger’s and Begg’s tests also showed that there is no publication bias.

Our meta-analysis showed that SS was negatively correlated with FCR, and the level of FCR decreased as the SS increased. Such a conclusion may be warranted, because the low level of FCR may be explained by the theoretical model of social cognitive processing (79) and clinical practice. A social cognitive processing model (79) emphasizes that a supportive social environment optimizes individuals’ cognitive processing of traumatic events (e.g., cancer experiences) (80), promotes psychological adjustment to the stressors, and generates positive perceptions of the disease, and thus reduces the level of FCR (80). Therefore, providing a supportive social environment for cancer patients is an important strategy to improve the levels of FCR in patients, which also reminds us that we should pay close attention to patients’ worries about cancer recurrence and emotional expression in clinical practice. Our results were consistent with findings from previous studies (35, 36). For example, Indonesian gynecological cancer survivors received social, emotional, spiritual, and even financial support not only from their family or close relatives but also from their neighbors and colleagues since Indonesians have a strong collective culture rooted in Chinese Confucianism (35). Correspondingly, Ashing et al. (36) found that Chinese women had a lower FCR than women from other Asian countries (e.g., Korea, Philippines, and Vietnam), which may be related to the fact that Chinese women might receive the greatest amount of support from their fellow Chinese and the community, which contributed to their lower FCR scores. However, Singh-Carlson et al. (37) noted that South Asian cancer survivors were ambivalent about receiving emotional support from family and community. These patients were sometimes reluctant to reveal their cancer diagnosis to their families and communities due to the pervasive stigma around cancer in their cultures and the prevailing belief of cancer as a death sentence (37). In turn, this occasioned feelings of isolation and depression among survivors, which heightened rumination and FCR. This was consistent with a study conducted in Iran in which the social circles of Iranian cancer patients tended to avoid giving information about cancer patients, contributing to misperceptions and high FCR levels in patients (38). However, Thewes et al. (39) found no correlation between SS and FCR among Australian breast cancer patients. These studies are among the numerous studies evaluating the interplays of the sociocultural dimension of FCR, suggesting that cultural background from different countries may account for FCR variation (43, 81), and different cultural groups may have distinctive communication, religious belief, SS, and coping mechanisms that contribute to the heterogeneity of FCR (30). One drawback is that although there is increasing evidence of the correlation between SS and FCR among cancer patients globally, a meta-analysis analyzing the relation of these two variables is yet to be published. Besides, although numerous empirical studies have shown a negative correlation between SS and FCR, pooled correlation coefficients could not be determined because of the lack of a meta-analysis exploring the link between the two variables. Therefore, the current study failed to compare the findings between China and other countries to evaluate the differences in the degree of the correlation between SS and FCR. Moreover, given that this study only focused on the Chinese population, future research can extend the scope of this research to cover other countries. Furthermore, a meta-analysis of the correlation between SS and FCR should be conducted between and among countries to validate the conclusions of this study. The impact of FCR on SS can be compared between high- and low-FCR-incidence countries.

Given the high heterogeneity identified in the analysis (*I*^2^ = 91% for the pooled *ß’*, *I*^2^ = 89% for the summary *r)*, meta regression, subgroup analysis, and sensitivity analysis were conducted to determine the reason for the heterogeneity. The results showed that in terms of study design, sample size, random sampling, region of China, follow-up, FCR measure, SS measure were not moderating factors (Tables 3, 4), while types of cancer were heterogeneous source. In addition, sensitivity analysis showed that study type were potential sources of heterogeneity.

Sample size did not have a significant effect on SS and FCR, pending future analysis in a larger number of study cases. Similarly, there was no significant effect on SS and FCR when using the random and non-random sampling methods, which might be only one study literature was included in the random sampling, the effect of sampling method on the correlation coefficient needs to be further investigated. Different regions also showed no differences in SS and FCR, but further studies were needed considering that the northern and southern regions in China may be related to the different living environments and social support network systems. Furthermore, consistent conclusions were reached regarding the covariate of follow-up. However, longitudinal follow-up studies can be used to examine changes in the trajectory of FCR and this has been mentioned in several meta-analyses (82–84). Lebel et al. (8) further proposed that FCR may show symptoms immediately after diagnosis and persist for several years. Ren et al. (43) found that the FCR exhibited dynamic changes and its development trajectory should be dynamically observed. Waters et al. (85) demonstrated that the connection between SS and FCR persisted for at least 6 months. Enhancing patients’ SS may lessen the severity of FCR. Future investigations should involve longitudinal designs to examine the association between SS and FCR at various time periods given the importance of longitudinal follow-up studies, which are currently uncommon in China and abroad when compared to the cross-section.

The moderating effect analysis revealed that types of cancer could moderate the relationship between SS and FCR (*P*^a^** = 0.047, Table 4), and the summary *r* coefficient were higher in the mixed-type group studies compared with other-type group studies and breast cancer group studies (summary *r*: –0.64 *vs*. –0.56 *vs*. –0.43, *p* < 0.01) (Table 4). Although the effect of types of cancer on FCR is not well-understood (86), several studies have found that breast cancer patients have the highest levels of FCR (17, 87). And in line with this published meta-analyses (82–84) showed that in the breast cancer group, the *r* coefficient correlation between chemotherapy/radiotherapy and FCR was significant. This was inconsistent with findings of our study and may be due to the following reasons. Firstly, it may be caused by the lack of literature in some groups. For example, there was only one study in the mixed-type group. Although other-type group had many studies; up to 21 studies, the literature for each cancer from other-type group was relatively small (three for nasopharyngeal, three for liver, three for colorectal, two for laryngeal, two for lung, one for glioma). Secondly, most of the current FCR-related studies focus on breast cancer patients (10, 88), as mentioned by Yang et al in a previous meta-analysis (82). Based on this background, people further investigated the effects on FCR from multiple other aspects (other factors besides SS), which in turn affected FCR to a certain extent, thus it is possible that the influence of breast cancer studies has somewhat generated a lower correlation value between SS and FCR. In addition, our results may be influenced by the Chinese traditional culture. Nevertheless, the results demonstrate that types of cancer may moderate the relationship between SS and FCR, which is a significant finding. Therefore, future research focusing on breast cancer should also pay attention to other types of cancer patients. Sensitivity analysis showed that after removing the study by Li et al. (32), heterogeneity decreased to 75%, suggesting that study type was a source of the heterogeneity because all studies investigating the relationship were cross-sectional, except that of Li et al. (32), which was a prospective cross-sectional study. In addition, as for family support and other support from PSSS scale, after excluding Li et al. (41), Lin (53), respectively, the heterogeneity was reduced to 72 and 83%, showing that these two studies were the main sources of heterogeneity. However, this study found that types of SS (actual SS, PSS, and others, *P^a^* = 0.581), source of actual SS (*P^a^* = 0.553, measured by SSRS), and source of PSS (*P*^a^** = 0.676, measured by PSSS) were not a significant moderator (Table 4), but the topic (type and source of SS) was known to be important to cancer’ SS and FCR (26). Future research should continue to examine the influence of SS type and different sources of each SS type on FCR in Chinese cancer survivors in a larger sample.

### Strengths and limitations

This study has provided several important theoretical concepts. First, it is the first systematic review and meta-analysis to quantitatively investigate the association between SS and FCR using *ß’* and *r* coefficients, and this is expected to improve our theoretical understanding of the association between SS and FCR. Second, the variables involved in the association between SS and FCR were assessed using a validated scale. Third, our study is evidence-based, and by clarifying the degree of correlation between SS and FCR, important insights into the correlation between SS and FCR among social workers and medical staff were revealed. However, this study has several potential limitations. First, we only included Chinese patients, and the results may not be generalized to other populations. Second, we only included articles published in English and Chinese, and thus some potentially high-quality data published in other languages could have been missed. Third, cross-sectional data can only demonstrate an association rather than offer causality. Three potential future research directions were identified in this study. First, current studies on SS and FCR are mostly cross-sectional, future research should adopt the experimental or longitudinal designs to explore causal associations. Second, SS and FCR are subjective psychological states, and thus qualitative and quantitative studies should be conducted to increase our understanding on SS and FCR to gain in-depth knowledge about the appropriate interventions for reducing cancer patient’ FCR. Third, larger sample covering multiple public hospitals should be included in future studies to increase the generalizability of results.

## Conclusion

In conclusion, this study found that SS was negatively associated with the degree of FCR among Chinese cancer patients, and the level of FCR decreased as the SS increased. Meta-regression and subgroup analyses revealed that types of cancer and study type were potential sources of heterogeneity, suggesting that these moderators of the association between SS and FCR should also be further investigated studied because they can be used to identify patients in need of treatment. However, types of SS (actual SS, PSS, and others), source of actual SS, and source of PSS were not significant moderators, but the topic (types and sources of SS) was known to be important to cancer patients’ SS and FCR (26), therefore future research should explore the influence of SS type and different sources of each SS type on FCR among Chinese cancer survivors using larger samples. In addition, even though our meta-analysis did reveal that SS was negatively related with the FCR, we were unable to ignore the possibility that in some cancer patients, FCR may have a negative impact on SS since people may isolate themselves or be less available to others or be more dissatisfied due to their distress. Therefore, large sample size and well-designed studies are needed in the future. Furthermore, given the longitudinal trend in the development of FCR and the subjective psychological state, longitudinal research and mixed research are needed to reveal the relationship between SS and FCR in detail.

## Data availability statement

The original contributions presented in this study are included in the article/Supplementary material, further inquiries can be directed to the corresponding authors.

## Author contributions

XL, CW, DB, and JG designed the study and main coordinators of the study. XL was the principal investigator, guarantor, and the one to write the essay with the support of JG, CH, CW, and DB. QY, MC, and WW carried out the study. XL, CW, and DB managed the literature searches and analyses. QY and MC responsible for the statistical and epidemiological support. All authors reviewed and approved the final version of the manuscript.
