# Supplementary material for: Relationship between social support and fear of cancer recurrence among Chinese cancer patients: A systematic review and meta-analysis
Source: Front Psychiatry. 2023 Mar 8;14:1136013. doi: 10.3389/fpsyt.2023.1136013 (PMC10031045; doi:10.3389/fpsyt.2023.1136013)

Appendix A: Systematic literature review search strategy in Pubmed

#1 "neoplasms"[MeSH]

#2 "malignancy"[Title/Abstract] OR "cancer"[Title/Abstract] OR "tumor*"

[Title/Abstract] OR "neoplasm"[Title/Abstract] OR "neoplasm"[Text Word] OR

"tumor*"[Text Word] OR "cancer"[Text Word] OR "malignancy"[Text Word]

#3 #1 OR #2

#4 "recurrence"[MeSH]

#5 "recurrence"[Title/Abstract] OR "relapse"[Title/Abstract] OR "progression"

[Title/Abstract] OR "exacerbation"[Title/Abstract] OR "return"[Title/Abstract] OR "return"[Text Word] OR "exacerbation"[Text Word] OR "progression"

[Text Word] OR "relapse"[Text Word] OR "recurrence"[Text Word])

#6 #4 OR #5

#7 "fear"[Title/Abstract] OR "worry"[Title/Abstract] OR "fear of cancer recurrence"

[Title/Abstract] OR "uncertainty"[Title/Abstract] OR "concern"[Title/Abstract]

OR "concern"[Text Word] OR "uncertainty"[Text Word] OR "fear of cancer recurrence"[Text Word] OR "fear"[Text Word] OR "worry"[Text Word])

#8 "social support"[MeSH]

#9 "social support"[Title/Abstract] OR "perceived social support"[Title/Abstract]

OR "perceived social support"[Text Word] OR "social support"[Text Word]

#10 #8 OR #9

#11 #3 AND #6 AND #7 AND #10

Appendix B: JBI critical appraisal checklist for studies

| Items | Yes | No | Unclear | Not applicable |
| --- | --- | --- | --- | --- |
| 1. Was the sample frame appropriate to address the target population? |  |  |  |  |
| 2. Were study participants sampled in an appropriate way? |  |  |  |  |
| 3. Was the sample size adequate? |  |  |  |  |
| 4. Were the study subjects and the setting described in detail? |  |  |  |  |
| 5. Was the data analysis conducted with sufficient coverage of the identified sample? |  |  |  |  |
| 6. Were valid methods used for the identification of the condition? |  |  |  |  |
| 7. Was the condition measured in a standard, reliable way for all participants? |  |  |  |  |
| 8. Was there appropriate statistical analysis? |  |  |  |  |
| 9. Was the response rate adequate, and if not, was the low response rate managed appropriately? |  |  |  |  |
|  | | | | |

| Quality assessment for the 37 studies in the current meta-analysis | | | | | | | | | | |
| --- | --- | --- | --- | --- | --- | --- | --- | --- | --- | --- |
| Study | Quality Item | | | | | | | | | |
|  | Item1 | Item2 | Item3 | Item4 | Item5 | Item6 | Item7 | Item8 | Item9 | Total |
| Zhong M S | Y | N | Y | N | Y | N | Y | Y | Y | 6 |
| Li Y F | Y | N | Y | N | Y | Y | Y | Y | Y | 7 |
| Guan H X | Y | N | Y | N | Y | N | Y | Y | Y | 6 |
| Zeng X X | Y | N | Y | N | Y | N | Y | Y | Y | 6 |
| Yuan W | Y | N | U | N | Y | Y | Y | Y | Y | 6 |
| Ye S M | Y | N | Y | N | Y | N | Y | Y | Y | 6 |
| Lin Y N | Y | N | Y | Y | Y | N | Y | Y | Y | 7 |
| Li S S | Y | N | Y | N | Y | N | Y | Y | Y | 6 |
| Cui L S | Y | N | Y | N | Y | N | Y | Y | Y | 6 |
| Deng Y Y | Y | N | Y | Y | Y | N | Y | Y | Y | 6 |
| Cheng Y | Y | N | Y | N | Y | N | Y | Y | Y | 6 |
| Chen Y F | Y | N | Y | N | Y | N | Y | Y | Y | 6 |
| Zhang Y | Y | N | Y | Y | Y | N | Y | Y | Y | 7 |
| Hu X C | Y | N | Y | N | Y | N | Y | Y | Y | 6 |
| Luo W X | Y | N | Y | Y | Y | N | Y | Y | Y | 7 |
| Zhao T Y | Y | N | Y | Y | Y | N | Y | Y | Y | 7 |
| Gao W J | Y | N | Y | N | Y | N | Y | Y | Y | 6 |
| Xu Z Z | Y | N | Y | Y | Y | N | Y | Y | Y | 7 |
| He B X | Y | N | Y | N | Y | N | Y | Y | Y | 6 |
| Liao L L | Y | N | Y | Y | Y | N | Y | Y | Y | 7 |
| Zhang H | Y | N | Y | Y | Y | N | Y | Y | Y | 7 |
| Zhai S P | Y | N | U | Y | Y | N | Y | Y | Y | 6 |
| Lai X L | Y | N | Y | N | Y | N | Y | Y | Y | 6 |
| Ma Y H | Y | N | Y | Y | Y | N | Y | Y | Y | 7 |
| Zhong X F | Y | N | Y | N | Y | N | Y | Y | Y | 6 |
| Li Y Y | Y | N | Y | N | Y | N | Y | Y | Y | 6 |
| Ye C L | Y | N | Y | N | Y | N | Y | Y | Y | 6 |
| Ren H | Y | Y | Y | N | Y | N | Y | Y | Y | 7 |
| Zhang Y | Y | N | Y | N | Y | N | Y | Y | Y | 6 |
| Guo H T | Y | N | Y | N | Y | N | Y | Y | Y | 6 |
| Zhou J J | Y | N | Y | N | Y | N | Y | Y | Y | 6 |
| Niu L S | Y | N | Y | N | Y | N | Y | Y | Y | 6 |
| Yue B | Y | N | Y | Y | Y | N | Y | Y | Y | 7 |
| Yu Z C | Y | N | Y | Y | Y | N | Y | Y | Y | 7 |
| Ren J L | Y | N | Y | N | Y | N | Y | Y | Y | 6 |
| Zhang R | Y | N | Y | N | Y | N | Y | Y | Y | 6 |
| Xing C | Y | N | Y | N | Y | N | Y | Y | Y | 6 |
| Abbreviations: Y, yes; N, No; U, unclear | | | | | | | | | | |

Appendix C: Quality assessment for the 37 studies in the current meta-analysis

Appendix D: Sensitivity analysis for the subgroup analysis

other support from PSSS


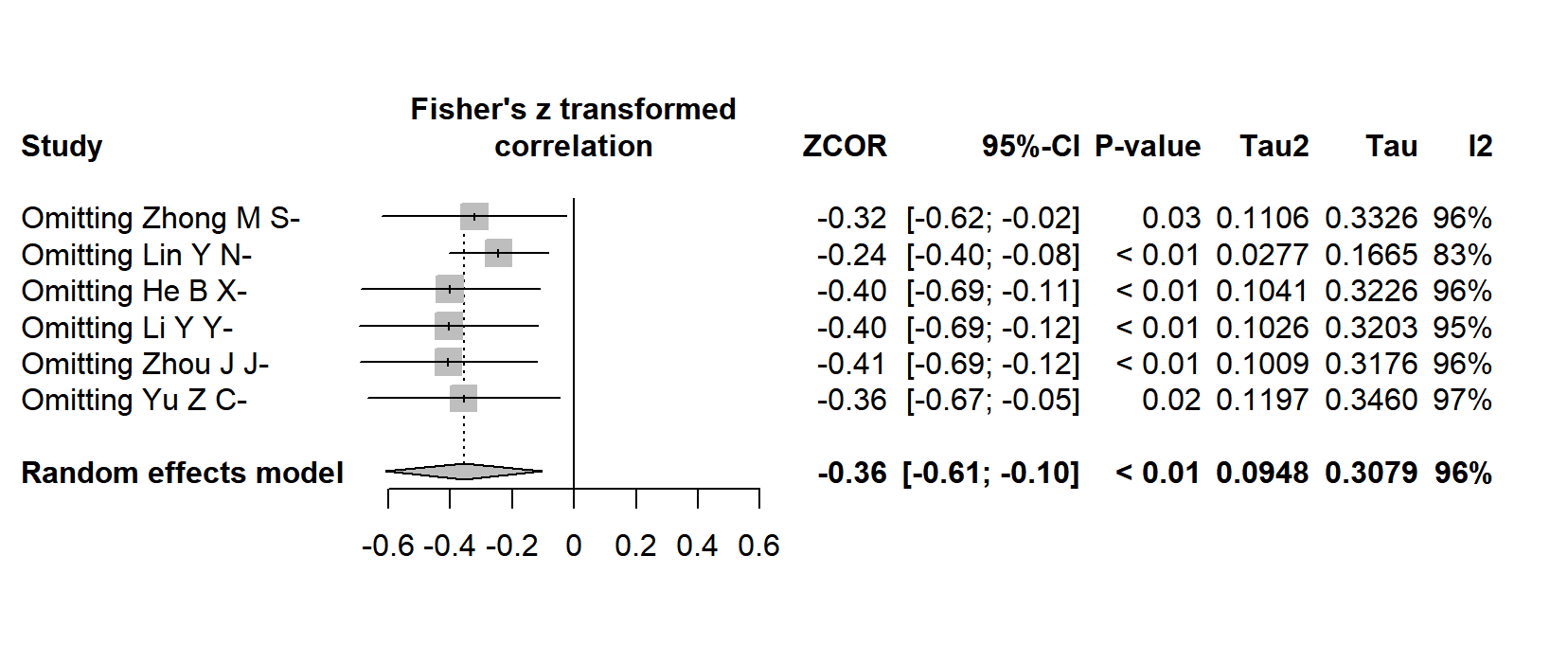


Family support from PSSS


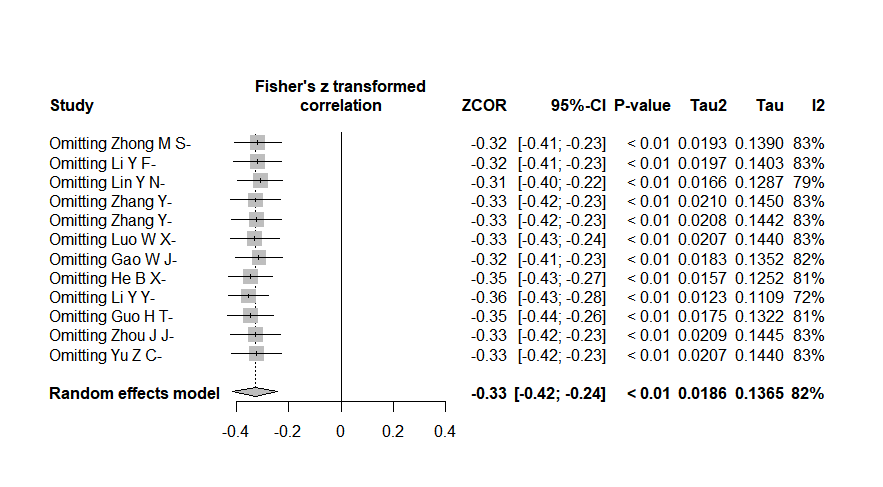


Follow-up


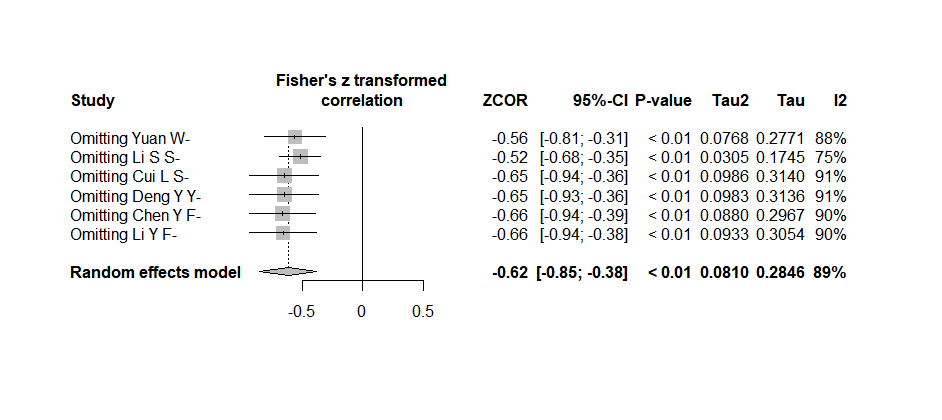


Appendix E: Sensitivity analysis for the β′ (A) and Correlations (B) between SS and FCR.


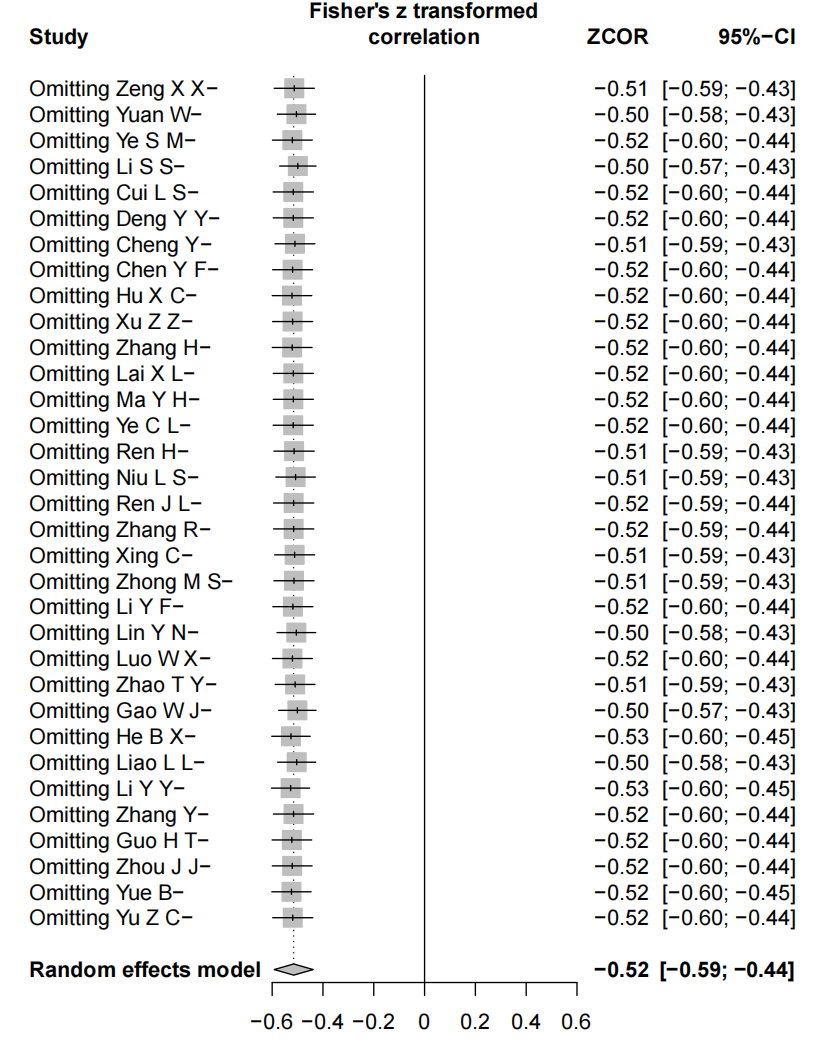

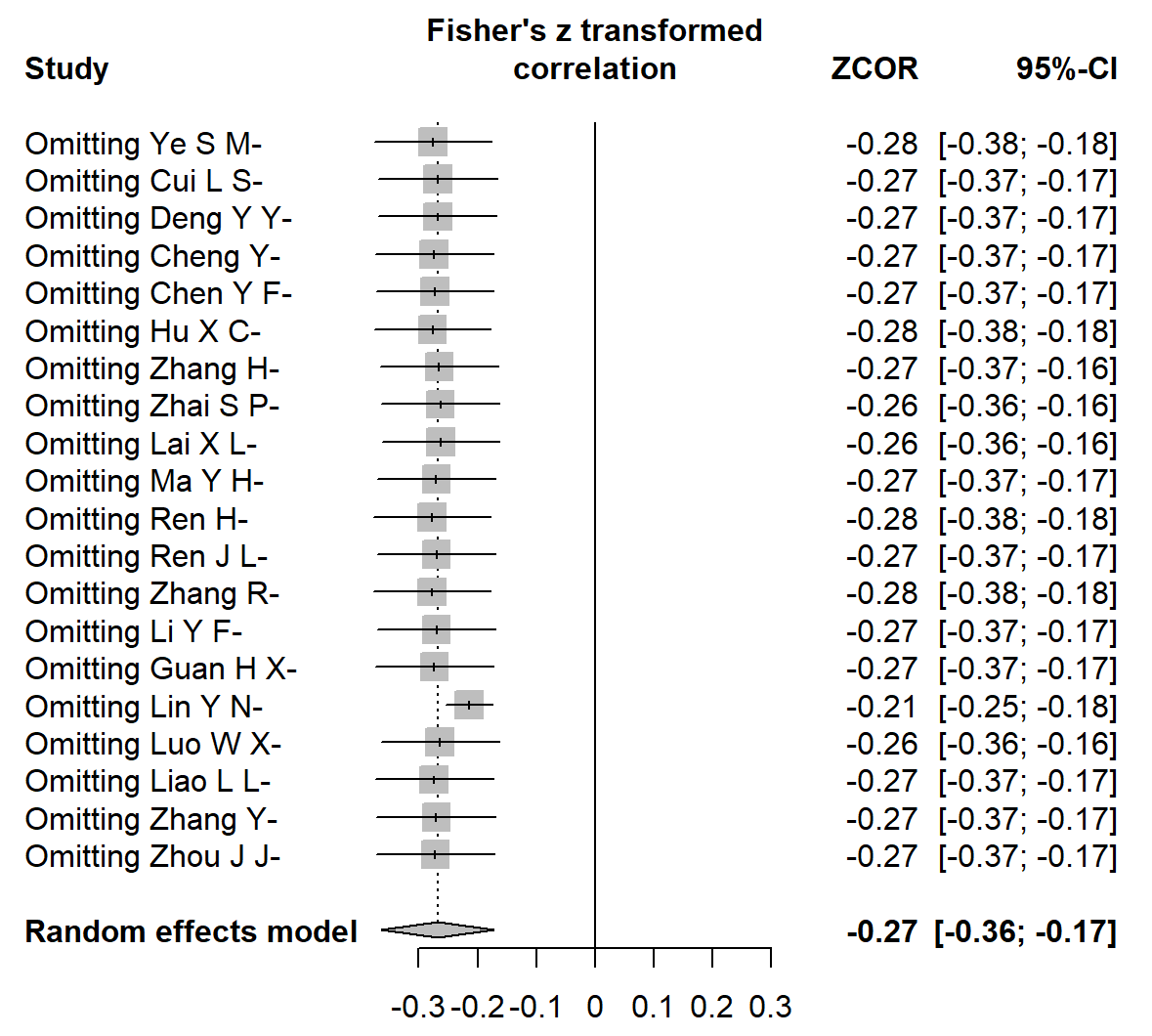


A

B

Appendix F: Funnel plots for assessing publication bias within studies, β′ (A) and correlation coefficient (B), respectively.


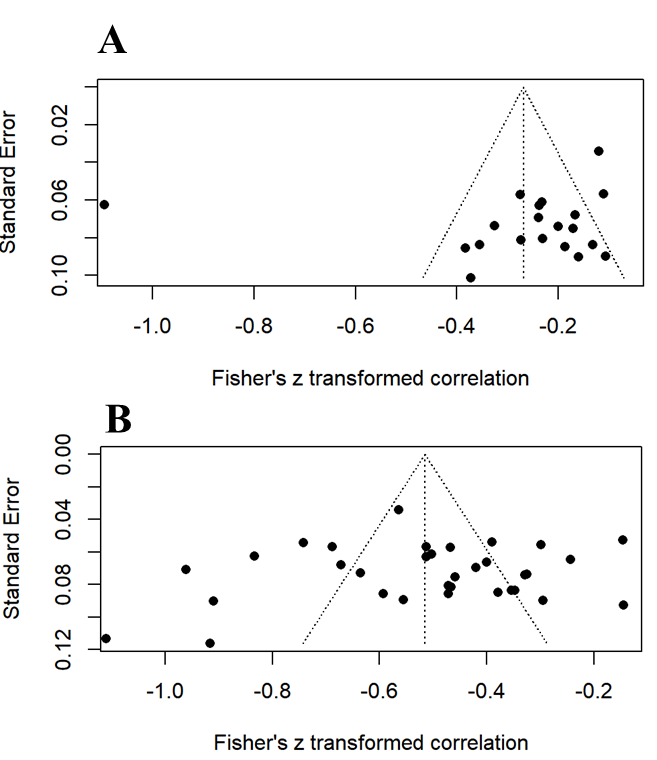

Supplement: Supplementary file 1 [file Data_Sheet_1.docx]
